# Impact of veterinary pharmaceuticals on environment and their mitigation through microbial bioremediation

**DOI:** 10.3389/fmicb.2024.1396116

**Published:** 2024-07-08

**Authors:** Humaira Saeed, Sudhakar Padmesh, Aditi Singh, Abhishek Nandy, Sujit Pratap Singh, Ravi K. Deshwal

**Affiliations:** ^1^Amity Institute of Biotechnology, Amity University Uttar Pradesh, Lucknow Campus, Lucknow, India; ^2^Faculty of Biosciences, Institute of Bioscience and Technology, Shri Ramswaroop Memorial University, Barabanki, India

**Keywords:** veterinary medicine, animal drugs, pharmaceutically active compounds, micropollutants, microbial electrochemical technologies, biological remediation

## Abstract

Veterinary medications are constantly being used for the diagnosis, treatment, and prevention of diseases in livestock. However, untreated veterinary drug active compounds are interminably discharged into numerous water bodies and terrestrial ecosystems, during production procedures, improper disposal of empty containers, unused medication or animal feed, and treatment procedures. This exhaustive review describes the different pathways through which veterinary medications enter the environment, discussing the role of agricultural practices and improper disposal methods. The detrimental effects of veterinary drug compounds on aquatic and terrestrial ecosystems are elaborated with examples of specific veterinary drugs and their known impacts. This review also aims to detail the mechanisms by which microbes degrade veterinary drug compounds as well as highlighting successful case studies and recent advancements in microbe-based bioremediation. It also elaborates on microbial electrochemical technologies as an eco-friendly solution for removing pharmaceutical pollutants from wastewater. Lastly, we have summarized potential innovations and challenges in implementing bioremediation on a large scale under the section prospects and advancements in this field.

## Introduction

**Figure 1:**
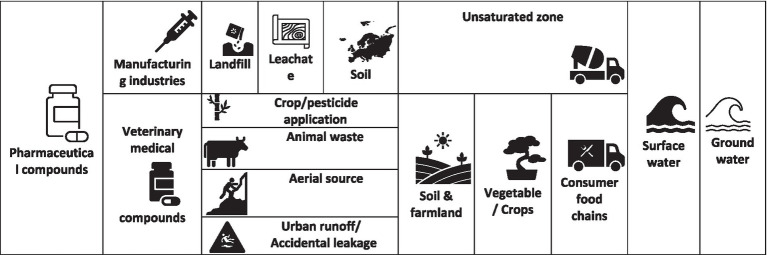
Graphical representation of entry of veterinary pharmaceutical into environment.

Pharmaceutical compounds that treat irregularities in animals are veterinary pharmaceutics. Veterinary drugs include biological products, hygienic goods, and products used to treat internal and external parasites as well as disease-transmitting vectors in animals. Veterinary drugs can also refer to any chemical or combination of compounds used in the diagnosis, treatment, or prevention of animal illness. Veterinary medicines are frequently used to treat illness and safeguard an animal’s health. Animals raised for food are also fed dietary boosting feed additives like growth promoters to accelerate their growth rates. When veterinary drugs are used in fish farms, for example, they are released into the environment both directly and indirectly when animal dung containing excreted products are spread on the ground. Every year, the production and use of pharmaceuticals increases significantly worldwide. The global consumption of different active compounds is expected to reach 100,000 tons or more per year. The concentration of antibiotics in wastewater is determined to be less than 1–150 μg/L, which may be increased further by nonmedical usage of antibiotics, such as agricultural, animal feeding activities, and so on (Singh and Saluja, 2021). According to long-term and multicentric research conducted by Klein et al. (2018), antibiotic consumption grew by 65% from 2000 to 2015, with a worrisome increase from 21 billion to over 35 billion specified daily doses. Concern over the destiny and impacts of these substances on the environment has increased along with this expansion. Low quantities of pharmaceutical chemicals are being found in ground and surface waters more often (Dar et al., 2019). Even though pharmaceutical ambient concentrations have been observed to date being far lower than the intended therapeutic dosages, it’s a global concern that these chemicals may have a negative effect on aquatic life and human health (Menzir and Adeladlw, 2022). Active raw components may be discharged during the production of an active pharmaceutical component and formulation of the completed drug product into the air, water, or land in the form of solid waste (Figure 1). Drugs end up in the environment during production primarily through the removal of functional pharmaceutical ingredients and coating equipment, mixing, compressing, as well as packaging tablets (Larsson, 2014; Singh and Saluja, 2021). Prior to the discharge of wastewater or sewage solids to surface waterways, effluent cleaning facilities, or ground, it is considered that the majority of drug residues are removed by biological and chemical degradation processes such biotransformation, mineralization, hydrolysis, and photolysis (Oluwole et al., 2020; Samal et al., 2022).

Veterinarians, animal owners, or domestic consumers are the main veterinary medication end users (Mutua et al., 2020). Veterinary pharmaceuticals may need to be disposed of at any point in their lifespan. Like with human drugs, it is probably reasonable to predict that some prescribed or over-the-counter veterinary medications may go unused by the intended patient. Disposal of veterinary pharmaceuticals should cautiously be taken into account by end users. Things like broken and expired veterinary medicines along with disposable boxes and packages, contaminated surgical instruments, gloves and bandages should also be taken into consideration (Rutala and Weber, 2015; Menzir and Adeladlw, 2022).

Previous studies on antibiotics degradation promisingly showed that living organisms such as bacteria, fungi, algae and plants are good for successful mitigation of these compounds. But not all the antibiotics can be degraded using a single microorganism because each veterinary pharmaceutical compound (e.g., antibiotics) varies in size from molecular to biological aspect like inoculum size, organism strain and different culture conditions. However, present mitigation processes like microbial fuel cell, saw dust and biochar have the hopeful potential in the possible removal of these pharmaceutics (Patwardhan et al., 2022). Many studies suggest that more efficient elimination of recalcitrant antibiotics can be achieved by combining different processes (Sodhi and Singh, 2022).

The aim of the present review is to understand the types and sources of veterinary pharmaceuticals, their fate in different environmental niches as well as their potential of causing antimicrobial resistance. We have also highlighted here the microbial way out for their removal through *in situ* and *ex situ* remediation or enzymatic degradation. The review also includes a brief on the upcoming cutting-edge technology of microbial electrochemical method for wastewater treatment, and the future challenges, making the paper a cornerstone resource for researchers in their work.

## Fate of veterinary pharmaceutical in environment

Veterinary pharmaceuticals can enter the environment through various pathways. Their fate involves processes such as degradation, transport and accumulation, impacting ecosystems and potentially leading to long-term environmental implications.

### Fate in manure and slurry

Large amounts of farmyard manure (animal urine and feces together with contaminated bedding material) and/or slurry (urine, feces, and washing-down water) are generated on livestock farms where animals are housed. Both can be used as an immediate organic matter supplement and fertilizer or kept in manure pits for later use (Velagaleti et al., 2003). Although there is no information on the fate of the degradation products, sulfonamides, aminoglycosides, lactams, and macrolides that have half-lives of 30 days are expected to be considerably degraded during manure/slurry storage. Ivermectin, tetracyclines, and quinolones, on the other hand, have longer half-lives and are therefore probably more persistent (Boxall et al., 2004). On the other hand, enormous amounts of farmyard manure and/or slurry are produced on livestock farms. Typically, this is collected and stored in manure pits for later application or applied to the ground right away. Slurry can be kept for a long time in storage. The time for storage of manure and slurry on an average is usually between 0 and 48 months with an average of 6 months (Gros et al., 2019).

### Fate in soil

Aerobic soil biodegradation is the primary method by which veterinary drugs are broken down in soils. Different medications degrade at different degrees in soil, and their t ½ can range from days to months. ‌In a study conducted by Topp et al. (2016), the half-life of azithromycin in soil was 12.8 days with the application of azithromycin at concentration 10 mg/kg soil. According to Liu et al. (2015), chlortetracycline was degraded in 4.7 days for 100 mg/kg of soil. A study conducted on soil from wheat field in china by Duan et al. (2017), reported that ofloxacin was degraded 85.6 and 87.3% with 10% compost and 10% compost with 2% of biochar. Oxytetracycline was degraded 86.6, 89.6, 93.7, and 95.4% at concentrations of 1, 3.6, 10, and 30 mg/kg of soil (Ma et al., 2016). Environmental factors that affect the degradation of veterinary medicines include temperature, pH, soil type, soil-organic carbon, nutrient conditions, and the presence of degrading bacteria that have evolved to destroy classes of medicine (Ingerslev et al., 2001). Other modes of chemical breakdown and depletion, such as soil photolysis and hydrolysis, may be significant as well, subject to the nature of the chemical. Since photodegradation is only likely to take place in the top layer of soil, farming practices including the depth and time of any plowing will have an impact on how long photodegradable compounds survive in the environment. In the topsoil layers, breakdown of byproducts of both photolytic and hydrolytic breakdown procedure may go through aerobic biodegradation (Ingerslev et al., 2001). A veterinary drug may deteriorate, leach into groundwater, be transferred to surface waters, or partition to soil particles once it has reached the soil. The weather at the time of application will have an impact on behavior to some extent. The ability of veterinary drugs to bind to soils varies greatly. As a result, there are significant differences in the mobility of several veterinary pharmaceuticals. Variations in soil-organic carbon cannot account for the large differences in distribution of chemical in various soils. In the case of carbadox, the highest known organic-carbon normalized sorption coefficient is around two times higher than the lowest documented value. The fact that several veterinary medications are ionizable around their pKa values while in the natural pH range of soils, helps to explain these significant discrepancies in sorption behavior. Therefore, drugs can exist in the environment as negatively charged, neutrally charged, zwitter ionic charged, and positively charged organisms. Depending on the species, interactions with soil may take the form of hydrophobic interactions, hydrogen bonds at the surface, electrostatic attraction, or surface bridging.

### Fate in surface waters

**Figure 2:**
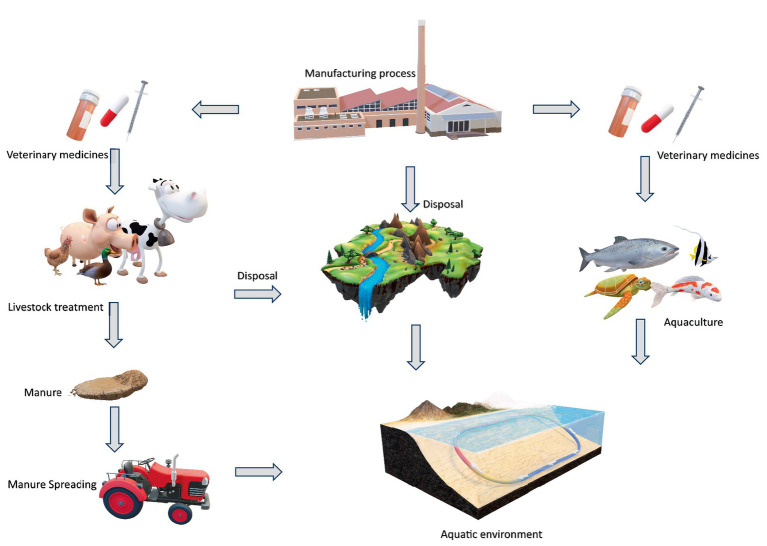
Entry of recalcitrant veterinary pharmaceuticals in aquatic environment.

Wastewater treatment plant effluent, are the main sources of contamination by veterinary medicines mostly antibiotic in the environmental compartments mainly surface and underground water (Ezzariai et al., 2018). Due to water shortages driven upon by pollution, urbanization, climate change, and regional droughts, wastewater is frequently utilized to irrigate agricultural land in arid and semi-arid countries. As a result, wastewater is now a useful resource for irrigating agricultural land. Veterinary antibiotics can enter into the ecosystem by way of untreated wastewater discharge into water bodies as lakes and rivers, livestock grazing on land that has been sprayed with manure and slurry, or direct cattle deposition. The pathway of recalcitrant veterinary pharmaceuticals’ entry into environment can be understood with Figure 2. Antibiotics have the potential to build up in soil and then be absorbed by crops when untreated wastewater or animal dung is utilized for agricultural irrigation or fertilization (Pan and Chu, 2017). Monensin concentrations of up to 183 μg/kg have been found in fertilized soils, which could be sources of contamination for surface and groundwater. Antibiotic ionophore values of up to 9 μg/L have also been found in these soils (Delgado et al., 2023). Another route is aquaculture, or fish farming, which often involves the use of antibiotics to prevent or treat bacterial infections in fish populations (Carvalho and Santos, 2016). Antibiotics used in fish farms can enter the surrounding water bodies directly through excretion from treated fish, uneaten medicated feed, or improper disposal of unused medications (de Ilurdoz et al., 2022). Veterinary pharmaceutical residues undergo biotic (biodegradation) or abiotic (hydrolysis or photolysis) processes in water, which can lead to their deterioration and/or transformation. During dry seasons, these degradation mechanisms are more effective because a rise in temperature encourages microbial activity and photolysis (Charuaud et al., 2019). When parent veterinary pharmaceutical compounds degrade, several transformation products may be produced, for example in enrofloxacin and marbofloxacin, photolysis of the fluoroquinolones results in the synthesis of 5 and 2 photo-transformation products, respectively (Sturini et al., 2010).

## The role of veterinary drugs in spread of antimicrobial resistance

The use of antibiotics serves as growth promoters in animal farming, such as cattle, pigs, and poultry, as well as to enhance feeding efficiency, particularly in the agriculture and livestock sectors (Cowieson and Kluenter, 2019). Some major veterinary antibiotics and their mode of action, source and proposed use in animal diseases are presented in Table 1. The increased use of all existing antimicrobials for the well-being of humans, animals, and agriculture results in their recurrent and frequent discharge into the environment and ecological systems (Nielsen et al., 2022).

**Table 1:** Veterinary pharmaceuticals, their mode of action and application in veterinary medicine.

**Mode of action**		**Antibiotics**	**Classification**	**Examples**	**Applications in veterinary medicine**
Cell wall synthesis inhibition	Beta lactam antibiotics	Penicillin	Natural	Penicillin G, penicillin V	**Ruminant illnesses** include anthrax, listeriosis, leptospirosis, clostridial, and corynebacterial infections, as well as streptococcal mastitis and keratoconjunctivitis **Swine illnesses** include erysipelas, streptococcus, and clostridial **Infections in horses** include tetanus, strangles, strep throat, and foal pneumonia **Infections in dogs and cats** include streptococcal and clostridial. UTI **Poultry infections** include necrotic enteritis, ulcerative enteritis, and intestinal spirochetosis
			Anti-staphylococcal	Methicillin, Oxacillin, Nafcillin	
			Aminopenicillin	Ampicillin, Amoxicillin	
			Broad Spectrum Penicillin	Carbenicillin	
		Cephalosporin	Cephalothin, Cefamandole, Cefataxime		
		Carbapenem	Primaxin		
		Monobactam	Aztreonam		
	Non-beta lactam antibiotics	Glycopeptides	Vancomycin, Teicoplanin, Avoparcin		Vancomycin is a “last resort” medication in human medicine, with limited applicability in animals Avoparcin is commonly used to promote development in chickens and piglets
Cell membrane disruption		Polymixin	Polymixin B, Colistin (Polymixin E)		**Cattle:** colibacillosis, salmonellosis in calves, and mastitis **Swine:** Neonatal porcine colibacillosis **In horses**, Klebsiella spp. can cause bacterial keratitis or metritis **Infections in dogs and cats** include bacterial keratitis, otitis externa, and skin infections
Protein synthesis inhibition	30 s subunit	Aminoglycosides	Gentamicin, Tobramycin, Amikacin, Streptomycin, Kanamycin		Toxic antibiotics in this family are used orally or topically to treat Enterobacteriaceae infections Less toxic aminoglycosides are used to treat severe sepsis infection of gram-negative aerobes
		Tetracyclines	Chlortetracycline, Oxytetracycline, Demethylchlortetracycline, Rolitetracycline, Limecycline, Clomocycline, Methacycline, Doxycycline, Minocycline		Tetracyclines are generally used to treat borreliosis, brucellosis (typically in conjunction with rifampin or streptomycin), chlamydia, ehrlichiosis, leptospirosis, listeriosis, rickettsiosis, and tularemia
	50 s subunit	Macrolides	Erythromycin, Tylosin, Spiramycin, Tilmicosin, Tulathromycin		Erythromycin is a preferred antibiotic for treating *Campylobacter jejuni* Tylosin and Spiramycin are used to treat Mycoplasma infections and promote growth Tilmicosin targets *Mannheimia, Actinobacillus, Pasteurella & Mycoplasma*
		Chloramphenicol			It may be considered for several anaerobic diseases in companion animals, such as significant eye infections, prostatitis, otitis media/interna, and salmonellosis		
		Lincosamide	Clindamycin, Lincomycin, and Pirlimycin		Clindamycin is highly effective against anaerobic bacteria Lincomycin is commonly used to prevent and treat dysentery & mycoplasma infections In cattle, pilrimycin is used as an intramammary infusion for mastitis
		Streptogramins	Virginiamycin		Used largely as a growth promoting for livestock but has also been used to prevent laminitis in horses
Nucleic acid synthesis inhibition	DNA topoisomerase	Fluoroquinolones	Enrofloxacin, Ciprofloxacin, Danofloxacin, Difloxacin, Ibafloxacin, Marbofloxacin, Pradofloxacin, Orbifloxacin		**Ruminant diseases** include acute *respiratory illness, infections with E. coli, Salmonella, Mycoplasma*, mastitis, and conjuntivitis **Treatment for swine infections** caused by *Mycoplasma hyopneumoniae, Actinobacilluspleuropneumoniae, Escherichia coli,* and *Pasteurellamultocida* **For dogs and cats**, it is for prostatitis, mastitis, rhinitis, pyoderma, otitis, wound infections, peritonitis, osteomyelitis, and soft tissue infections
	RNA polymerase	Rifamycin	Rifampin, Rifabutin, Rifapentine		Rifampin is used as a first-line oral drug treatment for tuberculosis in humans
	Antifolates	Sulfonamides	Sulfadiazine, Sulfamethoxazole, Sulfadoxine		Act synergistically (and becomes bactericidal) in combination with diaminopyrimidines. (trimethoprim)
		DHFR inhibitor	Trimethoprim, Aditoprim, Baquiloprim, Ormetoprim		Act synergistically (and becomes bactericidal) in combination with sulfonamides

The emergence of antibiotic resistance in every organism is the primary failure of all antibiotics and other pharmaceutical companies. Human and animal health is put at risk due to horizontal gene transfer (resistance) in the bacterial population (Kivits et al., 2018). The antibiotic resistant genes and antibiotic resistant bacteria reduce the curative ability of antibacterial substances toward human and animal infections. As a result, the residues of antibiotics in surface water have the potential to disrupt essential bacterial cycles/mechanisms/processes that are critical to aquatic balance, agricultural balance, and animal productivity (Ribeiro et al., 2018). Secondary sources include wastewater treatment plant effluents, sewage leaks, and agricultural waste, as these chemicals are not completely metabolized and may escape decomposition. Antibiotics have been found in hospital wastewater and wastewater treatment plant effluent, biosolids from wastewater treatment plants, soil, surface and groundwaters, sediments, biota, and drinking water (Barancheshme and Munir, 2018; Williams and Kookana, 2018). Antibiotic overuse, underuse, and abuse are shaping and causing novel incidences of microbial resistance, particularly among bacterial species. The logic for this scenario is long-term antibiotic exposure at low concentrations. Antibiotic resistance is characterized by a rise in the minimum inhibitory concentration of antibiotics against microorganisms. It is a process by which bacteria may withstand the stress of antibiotics. Antibiotic resistance can be carried out by microbes by evading drug-target interactions, efflux of antibiotics from the cell (efflux pump TolC in *E. coli*), and modification of antibiotics and their metabolites by enzymes (e.g., ADP-ribosyl transferases and glycosyl transferases). These antibiotic modifying enzymes can transfer functional groups that covalently change the antibiotics via acetylation, phosphorylation, adenylation, nucleotidylation, ribosylation, and glycosylation with the help of a co-substrate, ATP (Camotti Bastos et al., 2018).

These implications can result in changes to cell surface receptors, redox processes, hydrolysis, and other such aspects, all leading to MDRS. Under strong antibiotic stress, several types of resistance genes are activated, which eventually becomes a part of regular bacterial machinery and hence generates resistant enzymes even under control environments. The antibiotic fosfomycin, which is used to treat multi-drug resistant bacterial infections, is an active antibiotic against *E. coli, Staphylococcus aureus, Streptococcus pneumonia, Enterococcus faecalis*, and *E. faecium,* but it is less effective against *Klebsiella pneumonia* and *Enterobacter cloacae*, this lower susceptibility is attributable to mutations in the GipT and UhpT transporters, as well as changes in the antibiotic target MurA enzyme and antibiotic modification by proteins FosA, FosB, or FosX (Lucas et al., 2018; Scortti et al., 2018). Veterinary pharmaceutics like lincomycin and ofloxacin have high risk at the concentrations detected in effluents and medium risk in their receiving water bodies (Liu et al., 2023), resulting in potential hazard to the healthy aquatic ecosystem. Antimicrobial resistant bacteria have grown excessively due to unrestricted practice and for their mitigation, use of alternate strategies other than antibiotics are being explored extensively, like plant based natural compounds (Mathur et al., 2013; Singh et al., 2016), probiotic strains (Rastogi et al., 2021) or use of bacteriophages (Singh et al., 2022; Padmesh et al., 2023).

## Biodegradation of veterinary medicine

Antibiotics can undergo both biological and non-biological degradation in a variety of settings, to differing degrees. Photolysis, hydrolysis, oxidative degradation, and ionizing radiation degradation are examples of non-biological degradation, whereas microorganisms, algae, and plants are examples of biodegradation in the natural environment (Li et al., 2019).

Natural biodegradation greatly contributes to the breakdown of antibiotics; bacteria, algae, and plants play a major role in this process. Antibiotic pollution can be made harmless by microorganisms by altering the structure and physicochemical characteristics of antibiotics and breaking down antibiotic residues from macromolecular compounds to small molecule compounds and finally to H_2_O and CO_2_. Numerous microbial species, including photosynthetic bacteria, lactic acid bacteria, actinomycetes, yeast, fermentation filamentous bacteria, *Bacillus subtilis,* and nitrated bacteria, have been found to be capable of breaking down antibiotics in the environment. Tetracycline was reported to be 78% destroyed by a yeast strain that was obtained from the pharmaceutical sewage plant’s discharge sample (Zheng et al., 2022). It has been discovered that crop plants (*Cucumis sativus, Lactuca sativa*, and *Phaseolus vulgaris*) may convert approximately 25% of stored enrofloxacin into ciprofloxacin through metabolism (Migliore et al., 2003). In a study conducted by Baena-Nogueras et al. (2017) it was reported that half life of sulfamethazine after biodegradation was 24 days in fresh water and that of Sulphamethoxazole was 13 days post biodegradation in sea water (Baena-Nogueras et al., 2017). Fluoroquinolone was reported to have half life of 10.4 days in river after biodegradation (Caracciolo et al., 2018). Koba et al. (2017) reported the half life of Trimethoprim in soil after biodegradation 13–84% of the initial concentration was degraded. The primary environmental factors that impact the microbial breakdown of antibiotics include temperature, pH, oxygen concentration, and the type of environment. Strong specificity and low cost allow microbial breakdown a feasible remedy for antibiotic contamination.

## The microbial way out

Veterinary pharmaceuticals can be difficult to treat due to their enormous amount, complex and extremely durable chemical structure, and harmful nature (Kaczala and Blum, 2016). Physical and chemical remediation methods, such as coagulation/flocculation, membrane filtration, adsorption, ion extraction and photodegradation are currently available (Mahmood et al., 2022). However, even sophisticated oxidation procedures such as the use of ozone, hydrogen peroxide, and UV radiation are not always appropriate as they are expensive and may cause secondary pollution (Cuerda-Correa et al., 2019). Table 2 below summarizes the advantages and disadvantages of traditional methods of antibiotic removal from environment. The rising detection of pharmaceuticals and their metabolites in the environment highlights the need for more efficient and low-cost remediation approaches such as bioremediation (Letsoalo et al., 2023).

**Table 2:** Pros and cons of traditional antibiotic removal methods.

Physicochemical techniques	Process	Pros and cons	References
Ionizing radiation	Ionizing radiation aka radioactivity causes electron removal from atoms and molecules. Therefore, it causes oxidation and breakdown of molecular bonds resulting in dissociation of chemical structure	Since Ionizing radiation does not use chemicals and requires mechanical tools, it does not pollute the environment the way different chemicals do. Radiation gives upper hand in treating harmful vet products since it can treat them by oxidizing reduction as well	Chu et al. (2021)
Electrochemical oxidation	Electrochemical oxidation is a waste material treatment technique that uses electrical current along with different oxidizing chemical compounds which generates hydroxyl radicals or oxidizing species	There is no sludge produced during this operation. In comparison to Fenton’s procedure, it is a more expensive method	Miyata et al., 2011
Advanced oxidation process (AOPs)	Antibiotics and their metabolites are eliminated using AOPs because they are hard to break Hydroxyl radical with a much larger redox potential than previous approaches are produced. Contributes to the oxidation of organic molecules. The usage of ozone, hydrogen peroxide, or the combination of metallic/semiconductors produces free radicals	Fewer refractors’ byproducts are created because intermediary metabolites are frequently deadlier compared to original molecule	Kurt et al., 2017
Fenton oxidation	Fenton oxidation is a sophisticated technology that is in high demand for antibiotic elimination. A radical process between Fe^2+^ and H2O2 produces free hydroxyl ions (OH*). When Fe^2+^ combines with an excess of OH-and H_2_O_2_, Fe^3+^ is produced	Fenton’s technique is mostly used to remove antibiotics which works by cell wall inhibition The downside of utilizing this procedure is the production of sludge due to the presence of iron molecules	O’Dowd and Pillai (2020)

## Bioremediation

**Figure 3:**
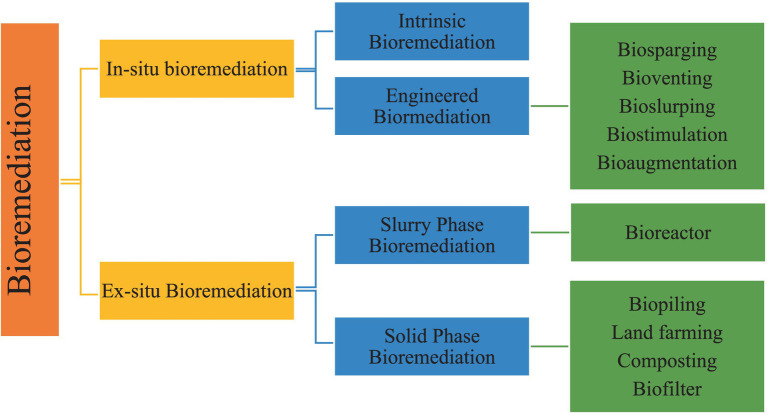
Schematic representation of Bioremediation and its classification.

The use of organisms that occur naturally or genetically designed microorganisms (bacteria and fungus) to consume and break down contaminants in contaminated media such as water, soil, and sediment is known as bioremediation or biological remediation (Singh and Singh, 2022). The definition of bioremediation according to USPEA (United States Environmental Protection Agency) is “an engineered technique that transforms environmental conditions (physical, chemical, biochemical, or microbiological) to promote microbes to destroy or detoxifying organic and inorganic contaminants in the environment.” The process is a well-established technology that has been used for decades to effectively degrade various forms of complex molecules, like chlorine-based solvents or hydrocarbons from petroleum in groundwater and soil. Bioremediation is broadly classified into two categories namely *in-situ* and *ex-situ* bioremediation (Figure 3).

## Microbial bioremediation

Antibiotic degradation is aided by microorganisms found in soil, sewage treatment facilities, and surface water (Patel et al., 2019). Bioremediation by a microbe is an attribute to a special metabolic potential possessed by the organism. Oxidation is a predominant and essential pathway for antibiotic degradation as well as photolysis, characterized by the degradation of the compound upon absorption of light energy, is also another significant mechanism in the breakdown of antibiotics. However, the success of degradation processes is dependent on the chemical nature of the pharmaceutical compound. For instance, tetracycline is smaller than cephalexin and thus more easily broken down by photolysis and UV (Azimi and Nezamzadeh-Ejhieh, 2015), whereas fluoroquinolones are resistant to hydrolysis but susceptible to UV. A number of microbes from all three major categories of bacteria, fungi and algae have been explored extensively for antibiotic bioremediation. For example, members of three prominent groups of filamentous fungi, the ascomycetes, basidiomycetes, and zygomycetes have been prominent in breaking down pollutants. However, it’s the white rot fungi that has been found to be most promising in pharmaceutical bioremediation (Amobonye et al., 2023). The major categories of microorganisms *viz.* bacteria, fungi and algae employed for pharmaceutical bioremediation (Table 3) are discussed below.

**Table 3:** Microorganisms and their optimum process conditions for antibiotic bioremediation.

Type	Name of organism	Veterinary medicine targeted	References
Bacteria	*Stenotrophomonas* sp. WA5 *Alcaligenes* sp. MMA	Amoxicillin	Yang et al. (2020) Bhattacharya and Khare (2022)
	*B. subtilis* 1556WTNC	Cephalexin, Ceftaroline, Ampicillin, Amoxicillin	Al-Gheethi et al. (2014)
	*Bacillus cereus*	Ampicillin	Anusha and Natarajan (2020)
	*Bacillus firmus* *Bacillus cereus* *Rhodococcus rhodochrous* *Rhodococcus equi* *Alcaligenes faecalis* *Acinetobacter* sp. *W1* *Bacillus licheniformis ATCC 14580*	Sulfonamides	Chen and Xie (2018) Wang and Wang (2018) Islas-Espinoza et al. (2012)
	*Lelliottiaaquatilis*	Amoxicillin	Zhang et al. (2020)
	*Stenotrophomonas maltophilia* DT1 *Klebsiella* sp. SQY5	Tetracycline	Leng et al. (2016) Shao et al. (2019)
Fungi	*Penicillium restrictum*	Sulfamethoxazole, Erythromycin	Fakhri et al. (2021)
	*Penicillium oxalicum*	Diclofenac	Kasonga et al. (2019)
	*Aspergillus niger* *Aspergillus luchuensis*		Kasonga et al. (2021) Dalecka et al. (2020)
	*Gymnopilus luteofolius*	Lopromide, Carbamazepine, Diclofenac	Vasiliadou et al. (2016)
	*T. versicolor*	Tetracycline, Chlortetracycline, Doxycycline, and Oxytetracycline	Suda et al. (2012)
	*T. versicolor*	Carbamazepine, Tetracycline Carbamazepine	Tormo-Budowski et al. (2021) Silva et al. (2019)
	*Chlamydomonas mexicana*	Carbamazepine	Silva et al. (2019)
Algae	*Eichhornia crassipes* *Chlamydomonas* sp. Tai-03	Ciprofloxacin	Sodhi and Singh (2021) Xie et al. (2020)
	*Scenedesmus obliquus*	Tramadol	Ali et al. (2018)
	*Nannochloris*	Triclosan, Diphenhydramine, Memantine, Trihexyphenidyl	Bai and Acharya (2016)
	*Scenedesmus obliquus*	Erythromycin	Wang et al. (2021)
	*Chlorella pyrenoidosa*	Cefradine, Cephalexin, Cefixime, Ceftazidime	Yu et al. (2017)
	*Chlorella protothecoides*; *Chlorella vulgaris*	Sulfamethoxazole, Amoxicillin, Trimethoprim, Ofloxacin, Clarithromycin	Ndlela et al. (2021)

### Bacteria-mediated bioremediation

In carrying antibiotics bioremediation, the bacterial population attains antibiotic resistant genes and some studies have shown positive correlation of antibiotic degradation potential with development of resistant genes (Zad et al., 2018). Bacteria are mostly employed in bioremediation. Bacteria utilized in bioremediation can live in harsh conditions; as they have an intrinsic ability to withstand and grow in adverse conditions (Ünal Turhan et al., 2019). Degradation of ciprofloxacin carried out by the mixture of *Bacteroidia* and *Proteobacteria* was proposed as well as dealkylation, deamination and C-F bond cleavage were some of the mechanisms by which ciprofloxacin was biodegraded (Liao et al., 2016; Sodhi and Singh, 2022). Chen and Xie (2018) proposed the microbial degradation of sulfonamide drugs in a sewage treatment plant. Several genera of microorganisms have been isolated from and characterized with efficient potential of sulfonamide drugs (Yang et al., 2021). The reactions by which these sulfonamide antibiotics degraded are oxidation, hydroxylation and acetylation. In a work by Zhang et al. (2020) using a model bacterium *Lelliottia aquatilis* and amoxicillin as a model β-Lactam antibiotic, improved detoxification of antibiotic wastewater was reported using a novel strategy of co-substrate combined with biological process. They have shown amplified activity of key enzymes, β-Lactamase, amidases, transaminase, and amide hydrolase, resulting in increased removal (95%) of amoxicillin with an increase of 60% from control (Zhang et al., 2020). A *Paracoccus* spp. isolated from sludge is studied for degradation of Penicillin G. Effective biotransformation of tetracycline by a novel strain *Stenotrophomona smaltophilia* DT1 (Leng et al., 2016) and *Klebsiella* sp. SQY5 (Shao et al., 2019) is reported.

In a recent study on bacteria mediated removal of antibiotics from wastewater sludge was studied for seven common antibiotics, namely oxytetracycline, tetracycline, chlortetracycline, amoxicillin, sulfamethazine, sulfamethoxazole andsulfadimethoxine. Twenty-four effective antibiotic degrading genera from both aerobic and anaerobic groups were identified from sludge, which had some well-known groups like *Acinetobacter, Bacillus, Burkholderia, Corynebacterium, Klebsiella, Mycobacterium, Pseudomonas*. However, they have also reported genera belonging to *Achromobacter, Acidovorax, Castellaniella, Comamonas, Dechloromonas, Gordonia, Novosphingobium, Pandoraea, Sphingomonas, Treponema and Xanthobacter* and some more as potential antibiotic degraders (Yang et al., 2020; Pathak et al., 2023). Batch fermentation using two different strains was found to be more effective than using a single strain. Additionally, these bacteria not only directly degraded antibiotics but also influenced the microbial community composition in the sludge, enhancing antibiotic degradation capacity (Yang et al., 2020).

### Fungi-mediated bioremediation

The biodegradation of veterinary medicines by fungal laccases enzyme, with the most remarkable outcomes obtained with *Trametes* laccases*. T. versicolor* laccases, for example, have been demonstrated to bioremediate the antibiotics tetracycline, chlortetracycline, doxycycline, and oxytetracycline (Suda et al., 2012). *Trametes polyzona*, another species of the same genus, was also shown to breakdown tetracycline, as well as several β-lactam and quinolone antibiotics (Lueangjaroenkit et al., 2019). In a study by Tormo-Budowski et al. (2021), *T. versicolor* immobilized on lignocellulosic substrate in a stirred tank bioreactor also substantially eliminated 16 PhACs in synthetic wastewater and those naturally occurring in veterinary hospital wastewater, with removal rates of 95.7 and 85.0%, respectively; however, it increased the general toxicity of wastewater matrices after treatment. The scientists then used a similar set up in a trickled bed bioreactor which led to significant removal of veterinary pharmaceutical compounds and detoxify the wastewater too (Tormo-Budowski et al., 2021). Another white rot fungus, *P. chrysosporium,* was used in a plate bioreactor and was used in both sequence batch and continuous modes to eradicate carbamazepine at substantial clearance rates ranging from 60 to 80%. The study also found that the bioreactor was successful even after running continuously for over 100 days. Apart from white-rot fungus, *Penicillium oxalicum*, an ascomycete, was employed for diclofenac biological remediation in a bench bioreactor, with cytochrome P450 enzyme activation playing important roles in its bioremediation endeavor (‌Olicón-Hernández et al., 2017). Similarly, a trickle-bed bioreactor based on various fungal biomass immobilized on rice husks was recently shown to eliminate 88.6 and 89.8% of PhACs in synthetic and real wastewater, respectively, and it was also demonstrated that adsorption was an important physical phenomenon in the trickle-bed bioreactor’s veterinary drug elimination effectiveness (Tormo-Budowski et al., 2021). The study conducted by Lucas et al. (2016), assessed the efficacy of a fungal treatment as a substitute veterinary wastewater treatment method for the removal of 47 veterinary antibiotics that fall into seven categories: β-lactams, trimethoprim, tetracyclines, macrolides, metronidazoles, sulfonamides, and fluoroquinolones. Following the fungal treatment, 77% of antibiotics were eliminated, which is more as compared to traditional treatment methods.

In this context, a wide range of fungal enzymes, including laccases, peroxidases, cytochrome P450 mixed function oxidases, lipases, and esterases, have been identified for their functions in degrading veterinary medicines. Fungal enzymes have been characterized as having extraordinary potential in the degradation of veterinary active compounds found in a variety of waste streams under different conditions. As a result, they provide a more cost-effective and ecologically friendly alternative to traditional treatment procedures (Vaksmaa et al., 2023). This amazing capacity has been attributed to their resilience, which enables them to breakdown complicated chemical structures into simpler and less hazardous compounds that may be digested further by other microbes (Rathore et al., 2022). The fungal enzymes have been found to aid in the biodegradation of nonpolar and poorly soluble pharmaceutically active veterinary compounds and other xenobiotics in organic solvents (Espinosa-Ortiz et al., 2021). These enzymes alter and detoxify medicines by reduction, oxidation, hydroxylation, dehalogenation, dehydrogenation, deamination, formylation, and other processes (Ferrando-Climent et al., 2015; Narayanan et al., 2022).

### Algae-mediated bioremediation

Aquatic environments are the principal producers of algae. Because of their short development cycle, high sensitivity to aquatic pollutants, and ability to begin stress response mechanisms, they can be employed as ecological indicators to eliminate pharmaceutical pollutants (Rodríguez Sánchez et al., 2015). Recently, algae-based techniques have been widely used for the treatment of antibiotic-containing wastewater, providing numerous benefits such as efficient CO_2_ fixation, low environmental impact, solar energy-driven activity, and the production of a potential raw material for the generation of biofuel or other high-value by-products (Xiong et al., 2021). For example, *Nannochloris* can entirely eliminate triclosan from water after 7 days of culture (Bai and Acharya, 2016), and it can efficiently digest diphenhydramine, memantine, and trihexyphenidyl, with average degradation rates of 88, 59, and 83%, respectively (Gojkovic et al., 2019). Organic pharmaceutical pollutants such as pesticides and antibiotics might be removed from groundwater using microalgae-mediated technology (up to 65%) (Ferrando and Matamoros, 2020). Similarly, Wang et al. (2021) introduced green alga *Scenedesmus obliquus* into wastewater to examine the erythromycin degradation routes. After 5 days of cultivation, the main mechanisms of erythromycin degradation in the microalgae-mediated system were biodegradation (including bioadsorption), hydrolysis, and photolysis, with biodegradation responsible for the highest proportion of removal (57.87%), followed by hydrolysis (34.13%), and photolysis (5%). It has previously been reported that *Chlamydomonas* sp. Tai-03 can entirely remove ciprofloxacin, with biodegradation accounting for up to 65.05% of clearance (Xie et al., 2020). According to Sutherland and Ralph, algae from the genera *Scenedesmus, Chlorella*, and *Chlamydomonas* are the species most explored and widely used for antibiotic bioremediation. Microalgae exhibit three primary pathways, bioadsorption, bio-uptake, and biodegradation for the bioremediation of pharmaceutical compounds, through which complex compounds undergo catalytic metabolic degradation. Furthermore, because microalgal cells are negatively charged and hydrophilic, lipophilic cationic chemicals show strong bioadsorption affinity with microalgae (Sutherland and Ralph, 2019). Ndlela et al. (2021) have demonstrated that *Chlorella protothecoides and Chlorella vulgaris* are effective in removing sulfamethoxazole (77.3 and 46.5%) and ofloxacin (43.5 and 55.1%) from samples containing 10 and 100 ppb concentrations, respectively.

### Enzyme-mediated bioremediation

Enzyme-mediated bioremediation is the process of cleaning polluted locations by using naturally existing enzymes found in microorganisms or plants to break down or eliminate harmful, unwanted, and resistant environmental contaminants (Karigar and Rao, 2011). Enzyme mediated bioremediation plays a significant part in the breakdown of antibiotics, which is crucial for the development of antibacterial resistance mechanisms. A correlation exists between the development of antibiotic-degraded enzymes, antibiotic-resistant genes, and antibiotic-resistant microorganisms. Study by Wencewicz (2019) shows that antibiotic resistance genes that express beta-lactamase enzymes and modify aminoglycosides existed long before the environment was exposed to synthetic antibiotics for medicinal purposes (Wang et al., 2020).

Enzymes are smaller than microbial cells, which makes it easier for them to come into touch with pollutants (Sharma et al., 2018). This helps with faster movement, more interaction with pollutants, and more focused, rapid, and efficient degradation or reduction to an acceptable or less dangerous form. The involvement of several enzymes (oxygenase, laccases, peroxidases, haloalkane dehalogenases, carboxylesterases, phosphodiesterase, lipases, and cellulases) in the breakdown routes of different pollutants is extensively described by Mansour et al. (2012). More operational condition flexibility is available when enzymes are applied rather than microorganisms or plants. They can be used in more severe environmental settings that are unsuitable for microbial populations, such as pollutant concentration, pH, temperature, and salinity (Cho et al., 2015). Compared to microbial or whole cell therapy, mass transfer limitation is significantly decreased with enzymatic treatment. Furthermore, enzymatic processes are simpler to manage than microbial remediation. Using enzymes for bioremediation is faster, less expensive, more accessible, and more focused than using plants or microorganisms. For microbial entire cells to develop at their best, air and nutrients must be introduced. Solvents and/or surfactants can improve bioavailability and immobilization (Demarche et al., 2012). From an enzymatic standpoint, it is also more practical than using whole cells. Additionally, compared to chemical and some forms of microbial remediation, enzyme-mediated biodegradation greatly minimizes the creation of hazardous byproducts. A further advancement in the miniaturization of analytical equipment with higher qualitative and quantitative quantification of chemicals of interest at site has been made by enzyme-based biosensors (Rocchitta et al., 2016).

### Microbial electrochemical technologies

Microbial electrochemical technologies stand out among cutting-edge water treatment methods as an effective and environmentally safe way to remove pharmaceutical pollutants in wastewater (Morris and Jin, 2007; McCormick et al., 2013). The advantageous features of microbiology, electrochemistry, and material science are combined in microbial electrochemical technologies, which offer viable methods for a variety of environmental engineering applications, such as resource recovery, wastewater treatment, bioremediation, and environmental monitoring. Microbial electrochemical technologies are often used in the bio-electro-Fenton process, microbial fuel cell, microbial electrolysis cells, and microbial desalination cell. METs have shown promise in recent years for the removal of stubborn pollutants and the rehabilitation of the environment because of their sustainable, effective, and energy-efficient characteristics (Xu et al., 2022). Microbial fuel cells (MFCs) harness the remarkable power of microbial communities to transform organic or inorganic compound oxidation into electricity through bio-electrochemical processes. This technology essentially taps into the natural metabolic activities of microbes, converting their energy-generating capabilities into a sustainable source of electric current (Gul et al., 2021; Nawaz et al., 2022). The primary source of METs, electrochemical active bacteria, can oxidize a variety of organic and inorganic materials found in sewage, organic waste, and wastewater used for dyeing. METs are a potential method for getting rid of drugs. Proteobacteria, Bacteroidetes, and Firmicutesemerge as the predominant organisms in mostmicrobial bioelectrochemical reactors (Hassan et al., 2021). *Pseudomonas* and *Klebsiella* are also a dominant genus in biocathodes for nitrofurazon degradation (Kong et al., 2017).

The first report on the use of METs for the removal of pharmaceutical pollutants, specifically antibiotics, has been reported by Wen et al. (2011). A specially designed two chambered microbial fuel cell was used for metronidazole degradation in wastewater treatment, and 85% removal of antibiotic was achieved within 24 h (Song et al., 2013). Still, several issues require attention. The main emphasis of future studies should be to support the environmentally friendly growth of METs during the treatment of pharmaceutical wastewater like constructing modeling tools to assess bio electrochemical systems. Other major concerns are to forecast treatment efficiencies of wastewaters containing pharmaceuticals under various operating conditions (Ortiz-Martínez et al., 2015) or combining METs with additional technologies, like a moving bed biofilm reactor (MBBR), to enhance the efficiency of pharmaceutical degradation (Chen F. et al., 2020; Chen M. et al., 2020). As pharmaceutical compounds can be highly variable in terms of their complex structure, continued research to determine their removal limits across different operational conditions becomes important. Some experimental electrochemical systems have shown promise of almost complete mineralization of antibiotic substances, e.g., microbial fuel cell coupled wetland constructs and microbial electrolytic cells and microbial fuel cell systems (Hassan et al., 2021). While significant progress has been made in understanding the antibiotic removal capacity of microbial electrochemical systems, it is still not enough considering the fast-growing environmental pollution.

## Prospects in advancement of microbial bioremediation

Microorganisms are at the forefront in bioremediation and environmental clean-up studies due to several documented benefits like ease of handling and cost-effectiveness. However, its robust implementation is still lacking, maybe due to insufficient scaling up of proven technologies and reluctance from industries. The special issue by Hlihor and Cozma (2023) highlights many such points as well as complex issues of application of microbial bioremediation in general. Therefore, bioremediation data must be vigorously searched in order to better understand degradative routes. New methods may be utilized to easily fit these data into simulation and numerical modeling, as well as data assembly, repositioning, exploration, and transmission, all of which need standard procedures. When combined with other physical and chemical processes, bioremediation can give a holistic strategy to remove various veterinary pharmaceuticals from the environment. In a recent review by Xu et al. (2024), the microbial enzyme mediated degradation of veterinary medicines is explored, with very high degradation efficiency in some cases (Bilal et al., 2019; Mathur et al., 2021). Approaches like immobilization and bioengineering techniques are extensively being explored with respect to microbial enzymes (Xu et al., 2024). Moreover, the application of microorganisms with genetic modification in the future to enhance bioremediation potential will be an effective technique. Studies have suggested that certain antibiotic resistance genes (ARGs), particularly those related to aminoglycoside modification and β-lactamase enzymes, have existed in environmental microbial communities prior to the use of synthetic antibiotics for medical purposes (D’Costa et al., 2011). This aspect needs to be explored more and understanding of naturally occurring genes can be identified. Over and above that, employing simultaneous gene transfer and multiplication of microorganisms with genetic modification will be a promising strategy.

Through newly found metabolic pathways, enzymes, genes, or operons; genetically designed microbes may become more efficient to bioremediate certain contaminants. Omics has set foot with great significance in the sphere of microbial remediation of veterinary pharmaceutical industry. While the field of genomics metabolomics, and proteomics in biological remediation help in the discovery of potential solutions to specific pollutants, the next frontier in bioremediation research is to find and compare gene and protein sequences that are successful at eliminating contaminants. Genetically modified organisms have the potential to clean up a wide spectrum of veterinary waste effluents and contaminated soil. In one such example, Crofts et al. (2018) have synthesized a genetically engineered *Escherichia coli* with ability to simultaneously express a β-lactamase and penicillin amidase or the penicillin utilization operon (*put*) operon. This modification enabled the strain to thrive by utilizing penicillin or benzyl penicilloic acid as growth substrates. This approach was utilized only after the genomic and transcriptomic studies of penicillin catabolism pathways unveiling the upregulation of β-lactamase, amidase, and the phenylactic acid catabolome (Crofts et al., 2018). Thus, there is a need for additional studies on the important genomic and proteomic codes of bioremediators found in nature. For this purpose, utilizing a systems biology approach also provides essential initial insights for the metabolic engineering of microbes, aimed at augmenting their bioremediation potential (Dangi et al., 2019). Understanding the variety and evolutionary links between various biological remediators can aid in the development of an even more effective bioremediation mechanism in the future. Similarly, for microbial electrochemical approach, it’s imperative to delve deeper into the dynamics of the interaction between these microorganisms and the electrodes, as well as between the electrodes and antibiotics. For instance, exploring modified electrodes could potentially boost gene expression linked to electron transfer mechanisms, thereby enhancing the efficiency of electron transfer involved in the degradation of antibiotics through redox reactions. So, with a good understanding of type of microbe, and metabolic redox reactions being carried out by them along with data on associated antibiotic-resistant bacteria and antibiotic resistance genes, we can innovate an effective solution for mitigating environmental antibiotic contamination (Kumar et al., 2019; Roy et al., 2022).

## Conclusion

The comprehensive management of veterinary pharmaceuticals requires a multi-pronged approach, combining regulatory measures with advanced treatment technologies. Microbe-based bioremediation, particularly using microbial consortia, shows promise as a sustainable and effective strategy. Accurately identifying the different biodegradation pathways that microorganisms use is necessary to improve comprehension. The latest developments in immobilization have made it possible to repurpose enzymes or whole cells that are involved in the breakdown of pharmaceuticals. The passage of intermediates and metabolites into and out of the immobilized support system is impacted by a few mass transfer limitations, though. Modern innovations like metabolic or genetic engineering and immobilization can greatly boost veterinary pharmaceutical biodegradation thanks to the identification of microbial degradation pathways. Numerous kinetic studies on the biodegradation of pharmaceuticals have been carried out. Therefore, more study that takes into account other process factors is needed to improve the estimation of the kinetic mechanism and degradation rate. However, further research and the development of hybrid technologies are essential to address the limitations and enhance the efficiency of existing methods.

## Author contributions

HS: Data curation, Formal analysis, Investigation, Methodology, Visualization, Writing – original draft. SP: Data curation, Formal analysis, Investigation, Methodology, Validation, Writing – original draft. AS: Data curation, Conceptualization, Supervision, Validation, Writing – review & editing. AN: Data curation, Formal analysis, Methodology, Writing – original draft. SS: Data curation, Resources, Writing – original draft. RD: Data curation, Formal analysis, Writing – original draft.
